# Medical Items

**Published:** 1893-04

**Authors:** 


### *MEDICAL ITEMS.*

THE next to the last item on page xxxiv in March number referred to the Three Chlorides of Messrs. Renz & Henry, Louisville, Ky.

ACCORDING to Dr. Harris, symphyseotomy has been done forty times since 1886. One mother has been lost and five children. There have been ten operations in America, three of which were in New York, two in Philadelphia, and one in Brooklyn.

DR. KEELY said in a lecture the other day that the great number of cures which he has effected could have been brought about only by "divine appointment." We observe that many of the numerous "institutes" in Chicago and other points are being closed up by the sheriffs. Presume this is also by divine appointment.

APROPOS of the commodious crinoline hoop-skirt which threatens to overtake us, a bill is introduced into the Minnesota legislature providing that "It will be unlawful for any person to manufacture or sell, or to offer for sale or use, or permit the manufacture, sale or use of any hoop-skirt, hoop-skirts or anything like thereunto, within the limits of Minnesota."

IT will be remembered that the Medical Department of the University of Texas began its career about two years ago with very large tuition fees. This fact caused some Texas medical students to seek their education elsewhere. Now the Board of Regents has practically abolished fees altogether. Fees will hereafter be only nominal, and a more liberal attendance of students will doubtless be forthcoming.

IN the Section on Therapeutics at the Pan-American Medical Congress, specialists and general practitioners are invited to contribute articles. Gentlemen desiring to read papers should notify the president of the section and forward abstracts by 10th of July in order that they may be translated into the three official languages and published in the program. The president of the section is Dr. Hobart A. Hare, Philadelphia.

---

THE Section on Dermatology and Syphilography of the Pan-American Medical Congress has been fully organized. In the list of honorary presidents, the leading cities of the Americas and the West Indies are represented. Secretaries are chosen from Canada to Brazil and the Sandwich Islands. Members of the Advisory Council are taken from the United States. Communications, notices of papers, etc., are to be sent to Dr. W. S. Gottheil, Secretary, 25 West 53d street, New York City.

---

DR. F. W. STOREY has removed from Clarksville, Red River county, Texas, to White Rock, Hunt county, Texas.

Dr. Wm. Thos. Coggin has removed from Keener, Ala., to Athens, Ga.

Dr. W. L. Patton has removed from Pensacola, Fla., to Mill town, Ga.

Dr. Roy J. Chappell has removed from Piedmont, Ga., to Columbus, Ga.

Dr. W. A. Priddy has removed from Divide, Ark., to Casa, Perry county.

---

THE third annual Convention of Military Surgeons will assemble in the amphitheatre of Rush Medical College, Chicago, August 8th. Papers on military surgery will be read by United States Army surgeons, and an interesting meeting is promised. Following are the officers: President, General Nicholas Senn, Chicago; Vice-President, Maj. Nelson H. Henry, New York; Second Vice-President, Lieut. Col. C. Meredyth Woodward, Tecumseh, Mich.; Secretary, Lieut. Col. Eustathius Chancellor, St. Louis; Corresponding Secretary, Lieut. Ralph Chandler, Milwaukee, Wis.; Treasurer, Col. Francis J. Crane, Denver, Colorado.

